# The Disruptive Impact of COVID-19 on the Utilization of Cancer Chemotherapy-Related Healthcare Assistance at the Principal Nationwide Referral Hospital in Kenya

**DOI:** 10.7759/cureus.50320

**Published:** 2023-12-11

**Authors:** Irene Mumbi Njunge, Faith Okalebo, Amanj Kurdi, Santosh Kumar, Susmita Sinha, Mainul Haque, Nihad Adnan, Johanna C Meyer, Brian Godman, Sylvia Opanga

**Affiliations:** 1 Pharmacology, Clinical Pharmacy and Pharmacy Practice, University of Nairobi, Nairobi, KEN; 2 Therapeutics, Strathclyde Institute of Pharmacy & Biomedical Sciences, University of Strathclyde, Glasgow, GBR; 3 Periodontology and Implantology, Karnavati School of Dentistry, Karnavati University, Gandhinagar, IND; 4 Physiology, Khulna City Medical College and Hospital, Khulna, BGD; 5 Karnavati Scientific Research Center, Karnavati School of Dentistry, Karnavati University, Gandhinagar, IND; 6 Pharmacology and Therapeutics, National Defence University of Malaysia, Kuala Lumpur, MYS; 7 Microbiology, Jahangirnagar University, Dhaka, BGD; 8 Public Health Pharmacy and Management, Sefako Makgatho Health Sciences University, Pretoria, ZAF; 9 Centre of Medical and Bio-Allied Health Sciences Research, Ajman University, Ajman, ARE; 10 Pharmacoepidemiology, Strathclyde Institute of Pharmacy & Biomedical Sciences, Glasgow, GBR; 11 Public Health Pharmacy and Management, School of Pharmacy, Sefako Makgatho Health Sciences University, Pretoria, ZAF

**Keywords:** breast cancer, national referral hospital, hospital care, consumption, kenya, shock, pandemic, sars-cov-2, chemotherapy, cancer

## Abstract

Background

Lockdown measures to reduce the outbreak of COVID-19 negatively impacted the administration of cancer chemotherapy globally; however, data from Kenya are limited. Researchers strove to address this information gap and assess chemotherapy trends before and during the pandemic at Kenyatta National Hospital (KNH), the most wide-ranging referral infirmary in Kenya, along with associated factors to provide future guidance.

Methods

Time series analyses and patient interviews were undertaken at the KNH Cancer Treatment Centre. Patient data were analyzed descriptively and inferentially. The average quarterly scores of chemotherapy-related patients from January 2019 to December 2020 were computed for the time series analysis.

Results

A total of 241 participants were recruited. Of the participants, 164 (68%) were female, and the mean age was 55. Breast cancer was the most typical cancer type. Independent risk factors for missed chemotherapy sessions were a considerable increase in travel costs, rescheduled appointments alongside difficulties in securing an appointment, comorbidities, and marital status. There was a decline in chemotherapy utilization before COVID-19, with a sharp drop at the pandemic's peak.

Conclusion

COVID-19 and associated measures did appreciably affect the treatment of cancer patients with chemotherapy in this developing country, with several factors contributing to this. Efforts should be geared toward continuity of services during future pandemics in developing countries to improve patient outcomes.

## Introduction

The coronavirus disease 2019 (COVID-19) was promulgated in China in December 2019 and was subsequently affirmed as a global pandemic by the World Health Organization (WHO) in early 2020 [1,2]. Subsequently, governments worldwide introduced several public health measures to contain its’ impact in the absence of efficient medications and vaccines [2-4]. The public health measures introduced across Africa included social distancing, contact tracing, curfews, and limiting face-to-face interactions. Alongside this, protection consists of wearing face masks, sanitizing, and washing hands [2,5-7]. In addition, there was the closure of educational establishments as well as the closure of ambulatory care clinics [8,9]. As a result, there was a considerable reduction in access to healthcare services among patients suffering from chronic illnesses and other conditions in the initial phases of the pandemic brought about by restrictions in movement, the reluctance of patients to attend hospital clinics for apprehension of contracting COVID-19, as well as job losses among patients especially in low- and middle-income countries (LMICs) affecting their ability to pay for travel costs to clinics alongside other essentials [10-16].

Any loss of income, coupled with an increase in travel costs, is especially important among LMICs where there can already be high patient co-payments for medicines and physician visits, exacerbating the catastrophic impact on patients and other members of their households infected with COVID-19. This will adversely impact seeking and funding care, particularly for managing non-communicable diseases (NCDs) [15,17-20]. There was also a general concern across countries that patients with NCDs received less attention than those with infectious diseases as resources within healthcare systems were shifted to tackle the pandemic [10,15,21,22]. This was seen, for instance, in the USA, where there was a significant reduction in patients admitted with acute cardiovascular illnesses, including heart failure, following the pandemic [23,24]. Across Europe, there was also a reduction in stroke patients seeking hospital care during the initial phases of the pandemic because of lockdown and other measures [10,25].

There are concerns generally with the therapeutic intervention of patients with NCDs because of movement control orders and other approaches with COVID-19 exacerbating the situation with NCDs, especially among LMICs, where NCDs such as diabetes and hypertension are still considerably underdiagnosed globally [15,21]. Healthcare services generally were also appreciably affected by the pandemic and its consequences among LMICs [3,13,26-30]. For instance, among LMICs, the pandemic resulted in shortages of medicines and increased lead times for their procurement as many LMICs relied heavily on developed countries and funded programs to supply these [3,31,32].

Cancer care is no exception, with appreciable concerns with identifying and initiating care, including those with breast carcinoma, across countries, including LMICs, especially during the early stages of the COVID-19 pandemic [33-39]. We have seen in many LMICs that the number of freshly diagnosed and reported cancer cases was reduced following lockdown measures. Alongside this, patients often presented with more advanced cancers when first seen by physicians due to reduced access to healthcare services during the pandemic [36,39-41]. Overall, the care of patients with cancer was disrupted in 88.2% of treatment centers globally due to the pandemic and associated activities [42]. This includes delays to surgery and radiotherapy [34,43,44].

The principal causes of service commotion to the care of malignancy cases in African countries were travel restrictions and clinic closures [45,46]. This, along with staff shortages and reduced access to effective treatments, including high costs of travel, appreciably impacted care delivery and outcomes across countries, including among African countries [34,42,44,46-49]. In the case of patients with breast malignancy, there was typically an increase in the prescribing of neo-adjuvant endocrine or chemotherapy therapy depending on the endocrine status of patients, in view of delays with surgery, as well as de-escalation, i.e., from IV to oral therapy, where this was possible [30,39,50-53]. More generally, a re-adjustment of treatment regimens, e.g., administering paclitaxel every 21 days rather than seven [30,39] and instigating telemedicine for follow-up care where this was practical and possible [54,55]. This is because approaches such as telemedicine require the necessary facilities and resources to work successfully. This was not always the case at the start of the pandemic in Africa, with many homes struggling, for instance, with internet and IT facilities [8].

Overall, the pandemic and associated measures increased concern with the management of patients with cancer across countries as cancer continues to be a leading cause of morbidity and mortality worldwide, with an estimated 18.1 million new cases globally in 2018 alone [56]. Currently, in Kenya, almost 7% of the national death rate is due to cancer. Consequently, cancer is now the 3rd prominent source of death in Kenya and is rising [45,56,57]. Many patients with suspected or actual cancer are usually referred to the national hospital, Kenyatta National Hospital (KNH), with a bed capacity of 2000 patients, in Kenya since regional hospitals currently have limited capacity and resources to offer comprehensive oncology services [45]. This is like several other African countries where specialist facilities for managing patients with cancer within the public system, including managing patients with cancer, are concentrated in specialist regional centers [58]. Following the implementation of government policies to contain COVID-19, notably lockdown measures, including curfews, a considerable number of patients across Africa, including Kenya, were unable to travel to specialist hospitals to seek care for their condition [46]. In addition, in Kenya and other African countries, there was also an increase in travel costs alongside a loss of income among patients due to the inability to work, further impacting the ability of patients to access services at specialist hospitals including KNH [59]. Other factors that are likely to have affected service delivery and uptake, including among African countries including Kenya, included an overwhelmed healthcare workforce and fear among cancer patients of contracting COVID-19 when attending hospital clinics [30,45].

However, currently, in Kenya, the magnitude to which the COVID-19 pandemic adversely affected care delivery for patients with cancer, including chemotherapy services, is unknown. This is a concern given the rising cancer prevalence and mortality rates across Kenya, like other African countries [58,60], and concerns generally with the extent of cancer services available in Kenya versus higher-income countries [58,61]. Consequently, this study sought to address this evidence gap by determining the trends of anticancer medicine utilization in KNH previously and ongoing the COVID-19 pandemic. Alongside this, the study aims to identify possible factors adversely affecting the uptake of these services. The findings can subsequently help to guide the establishment of future policies and procedures to improve the care of these priority patients in Kenya during future pandemics, building on current knowledge [45,58,61].

The findings of this study were presented as a poster at the International Conference on Pharmacoepidemiology in August 2023 in Copenhagen, Denmark. The abstract has been published in the Pharmacoepidemiology and Drug Safety Journal, Volume 31, 672-672, 2022.

## Materials and methods

**Figure 1:**
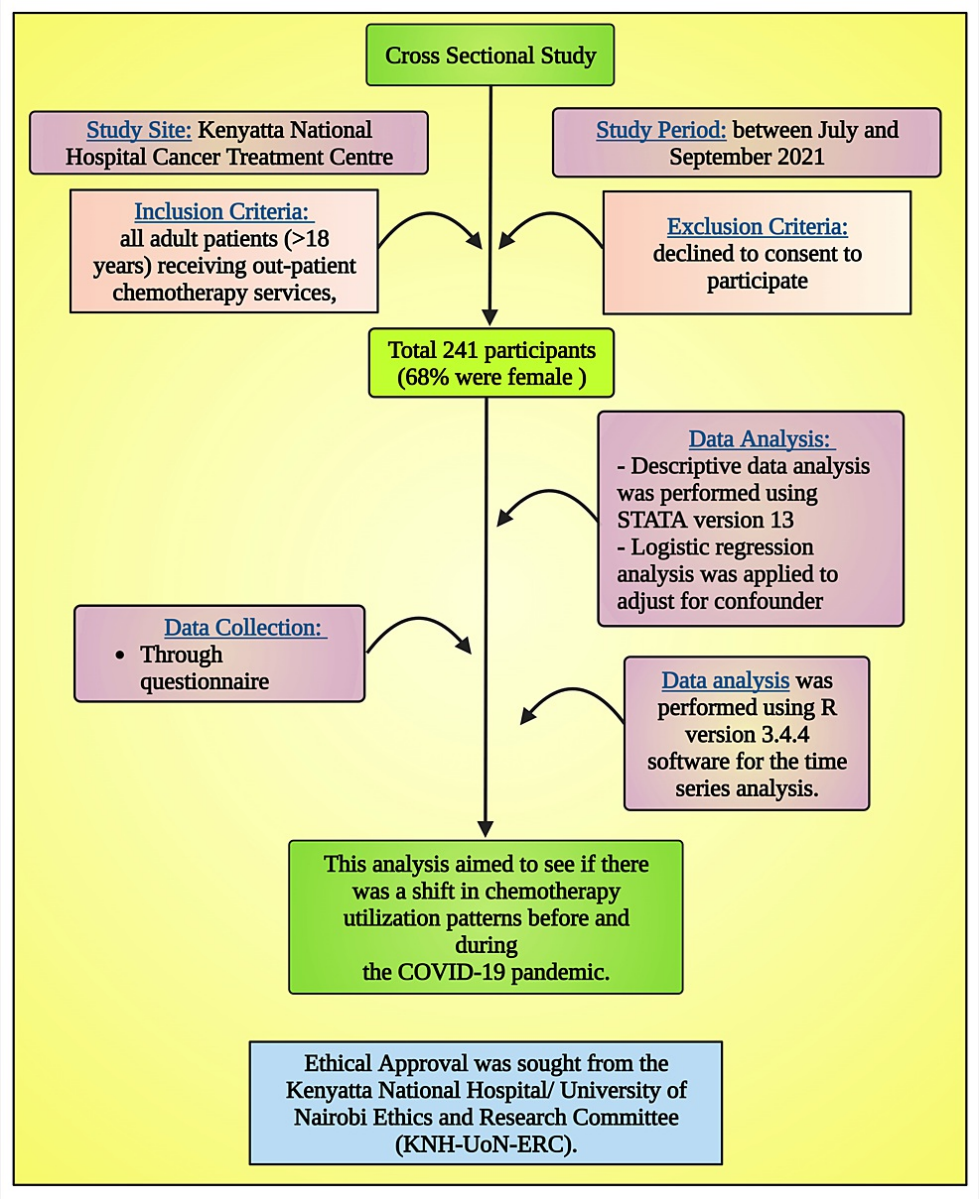
The schematic diagram of the study method of this research Notes: This figure has been drawn with the premium version of BioRender (https://biorender.com/; accessed on 8 November 2023) with license number UC262E9JTT. Image credit: Susmita Sinha.

The methodology (Figure 1) has already been described in detail [61], which is summarized below and builds on a recent review of the impact of COVID-19 on the management of patients with breast cancer, especially among LMICs [39].

Study site and duration

This cross-sectional research was undertaken between July and September 2021 among patients attending the Cancer Treatment Centre at KNH for chemotherapy. Before the pandemic, KNH was typically treating approximately 40 patients daily.

Study population, recruitment, and sampling

The Cochran (1977) formula was used to estimate the study sample size [62], building on Lou et al. (2020) [63], to arrive at an estimated sample size of 293 patients.

Adult patients (>18 years) receiving out-patient chemotherapy services, cancer in-patients, as well as patients receiving other treatment modalities, including radiotherapy, palliative care, and surgery, who all agreed to participate in the study were included. All others were excluded. A simple randomization procedure was used to recruit 14 patients daily [61], with each eligible patient given a unique serial number.

Data collection instruments including the retrospective data

Specific questionnaires were developed by the principal authors (IMN, SO, FO) using their considerable experience as no specific questionnaires were available in the literature. This included the data collection tool for the retrospective data regarding clinic attendance before the pandemic. This approach has been used before in similar African situations [3,4,8,64-67]. The questionnaire was subsequently pre-tested to enhance its validity, reliability, and robustness among 10 KNH Cancer Treatment Centre research participants. Based on the pre-testing results, the questionnaire was slightly modified, with a revised questionnaire subsequently submitted to the Ethics Committee at KNH [61].

Details on clinic attendance from January 2019 to December 2020 were abstracted by the principal investigator (IMN) from the patient’s records to provide baseline data [61].

Data collection and patient recruitment

Data were collected for the prospective study using the developed patient questionnaire by the principal investigator (IMN). Patients were asked in Kiswahili if they were unable to understand the questions in English [61]. Data collection occurred daily, before or during the patients’ chemotherapy sessions either in a specially designated room or in the chemotherapy infusion room [61]. A time series analysis was performed to evaluate the trends in chemotherapy utilization among patients before and during the pandemic [61].

Data management and analysis

Details of data management and analysis can be found in Njung’e IM's study [61].

Ethical considerations

Ethical approval was obtained from the Kenyatta National Hospital/University of Nairobi Ethics and Research Committee (KNH-UoN-ERC) with reference number P120102/2021, dated 19th July 2021. Respondents’ written informed consent was requested individually and voluntarily before answering the questionnaire, free from coercion. Each patient was provided with a comprehensive study description before giving informed consent, verbally and by signing the consent form. Those who could not sign provided their thumbprint.

There were immediate benefits to patients participating in the study, whereby those patients in need of assistance were referred to a social worker for further assessment. This included patients who showed evidence of defaulting their therapy through missing appointments, as well as those who verbally expressed that they had problems listed in the referral form. Alongside this, patients gained additional information from healthcare workers who directly attended to the participating patients and felt they were in need based on their professional opinion.

## Results

Key results are summarized in this section building on earlier comprehensive findings [61]. This includes two distinctive periods for the study, 2019 to 2020, i.e., before the COVID-19 pandemic and associated lockdown activities and in 2021 following the initial lockdown measures.

Patients' socio-demographic characteristics

A total of 241 patients were recruited into the study, with the majority being female (164, 68%). Most patients were above 50 years (156, 65%), with a mean age of 55 and a median age of 54 (21-90) years. Most recruited patients were married (180, 74.7%), and just under 70% (167, 69.3%) were unemployed. Nearly three-quarters of the recruited patients (180, 74.7%) were not residing in Nairobi, which had implications regarding their access to care during the pandemic and unemployment status.

Of the recruited patients, 47.3% had attained secondary-level education. Of the patients, 37.8% had a comorbidity, with the most prevalent being hypertension at 23.7%. Only 1.7% of patients stated that they consumed alcohol (Table 1).

**Table 1:** Details of study participants Note: All quoted parts denote reference [61].

Variable	Category	Frequency (n = 241)	Percentage (%)
Sex	Male	77	32.0
	Female	164	68.0
Age group	20-35 years	18	7.5
	36-50 years	66	27.5
	51-60 years	76	31.7
	60+ years	80	33.3
	Non-response	1	0.4
Age	Mean; median; range; IQR	55.0; 54.0; 21-90; 17	
Residence	Outside Nairobi	180	74.7
	Within Nairobi	61	25.3
“Marital status”	“Single”	53	22.0
	“Married”	180	74.7
	“Widowed’	8	3.3
Employment status	Unemployed	167	69.3
	Self-employed	53	22.0
	Employed	21	8.7
“Level of education”	“Primary and below”	96	39.8
	“Secondary”	114	47.3
	“Tertiary”	31	12.9
“Presence of comorbidity”	“No,” “Yes”	150, 91	62.2, 37.8
“Number of comorbidities”	“None,” “One”	150, 82	62.2, 34.0
	“Two”	8	3.3
	“Three”	1	0.4
Alcohol consumption	No	237	98.3
	Yes	4	1.7

Cancer-related characteristics

More than half of the recruited patients had either breast cancer (79, 32.8%), cervical cancer (36, 14.9%), or prostate cancer (33, 13.7%). The remaining 93 patients had other cancers, which included colon, nasopharyngeal, endometrial, lung, and esophageal cancer, as well as multiple myeloma and leukemia (Table 2).

**Table 2:** Types of cancers among study participants

Serial number	Cancer type	Frequency (n = patients)	Percentage
1	Breast	79	32.8%
2	Cervical	36	14.9%
3	Prostate	33	13.7%
4	Colon	19	7.9%
5	Nasopharyngeal	8	3.3%
6	Endometrial	6	2.5%
7	Lung	5	2.1%
8	Esophageal	5	2.1%
9	Multiple myeloma	3	1.2%
10	Lymphoma	3	1.2%
11	Leukemia	2	0.8%
12	Gastric, anal, osteogenic, renal, tongue	1 patient for each cancer	0.4% each
13	Others	37	15.1%
	Total	241	100

A total of 141 participants had their cancer staged, with 17 (7.1%) having stage I disease and 45 (22.4%) having stage II disease. Stage III and IV disease comprised 73 (30.3%) and 47 (19.5%) patients, respectively. Fifty (20.7%) patients had cancer cases that were not staged (I-IV).

Attendance at the chemotherapy sessions

Table 3 gives details of chemotherapy sessions before and during the pandemic.

**Table 3:** Details of chemotherapy sessions before and during the pandemic

Activity	Key findings
Initiation of chemotherapy sessions	85.1% of patients had their chemotherapy started before the COVID-19 pandemic. The remaining patients had their chemotherapy initiated during the COVID-19 lockdown restrictions.
Scheduling of chemotherapy sessions	63.9% (154 patients) had been scheduled for three to seven chemotherapy appointments when COVID-19 restrictions began in March 2020. 14.9% (36 patients) subsequently missed at least one appointment; however, 85.1% (205 patients) did not miss one appointment during the tight COVID-19 restrictions. 31 of the 36 patients who missed at least one appointment waited until lockdown measures were eased before visiting the hospital. Of the remainder, four patients received their therapy from a nearby facility, and one patient was referred to a different healthcare facility for assistance.
Oral cancer medicine availability	Of those participating patients on oral chemotherapy, 7.4% (5 patients) had no medicines and skipped their appointment, and 30.9% (21 patients) bought their prescribed medication from a chemist (community pharmacy). Overall, over half of the pertinent patients had sufficient stock that they used during the lockdown period.

Challenges encountered during the COVID-19 pandemic

The challenges encountered by participating cancer patients were more pronounced during the COVID-19 pandemic (Table 4). The principal challenge faced was the loss of income following the COVID-19 restrictions (202, 83.8%), followed by a considerable increase in travel fares (165, 68.5%). Subsequently, poor clinical outcomes in the event of healthcare workers’ strikes during the pandemic (123, 51.0%) were noted.

**Table 4:** Challenges encountered before and throughout the COVID-19 pandemic Note: All quoted parts denote reference [61].

Challenges	During COVID-19, n (%)	Before COVID-19, n (%)	p-value
Markdown of earnings	“202 (83.8%)”	“148 (61.4%)”	“<0.001”
Raise in transport cost	“165 (68.5%)”	“14 (5.8%)”	“<0.001”
“Poor clinical outcomes vs. health workers strike”	“123 (51.0%)”	“16 (6.6%)”	“<0.001”
“Long waiting time”	“120 (49.8%)”	“53 (22.0%)”	“<0.001”
“Living on the outskirts of Nairobi”	“112 (46.5%)”	“57 (23.7%)”	“<0.001”
“Rescheduled appointments”	“25 (10.4%)”	“11 (4.6%)”	“0.003”
“Others”	“24 (10.0%)”	“18 (7.5%)”	“0.377
“None”	“10 (4.1%)”	“55 (22.8%)”	“<0.001”
“Difficulty getting an appointment”	“7 (2.9%)”	“5 (2.1%)”	“0.754”

Association between different variables and missed clinic appointments

Table 5 highlights key findings from the bivariate analysis and challenges with missed appointments.

**Table 5:** Missed appointments and challenges during the COVID-19 pandemic and associated lockdown measures

Key finding	Analysis
Married patients vs. single patients	Married patients were 3.55 times more likely to miss an appointment than patients who were single: OR (95% CI): 3.55 (1.04, 12.07), p = 0.043.
Patients who called their physician during the pandemic	These patients were 2.95 times more likely to miss an appointment than those who did not call their doctor during the pandemic: OR (95% CI): 2.95 (1.04, 8.37), p = 0.041.
Key challenges associated with missed appointments during the pandemic	Key challenges included: Hikes in travel fares during the pandemic: OR (95% CI): 4.33 (1.47, 12.73), p = 0.008. Rescheduled appointments: OR (95% CI): 6.00 (2.46; 14.66), p < 0.001. Difficulty in getting to appointments: OR (95% CI): 16.37 (3.04, 88.09), p = 0.001. Living in the outskirts of Nairobi: OR (95% CI): 2.66 (1.26, 5.61), p = 0.010.

Independent predictors of missed appointments

Table 6 included independent predictors of missed appointments during the pandemic.

**Table 6:** Independent predictors of missed appointments KNH: Kenyatta National Hospital.

Key finding	Analysis
Marriage status	Married patients in this research were 6.31 times (95% CI: 1.36, 29.23; p = 0.019) more likely to miss an appointment than their single counterparts
Comorbidity	Patients with comorbidity 2.46 times more likely to miss an appointment compared with those with no comorbidities (95% CI: 1.05, 5.75; p = 0.038)
Increase in travel costs to KNH	Participating patients who faced an increase in travel costs to KNH during the pandemic were 4.56 times more likely to fail to keep their appointments (95% CI: 1.35, 15.47; p = 0.015
Appointments re-organized/re-scheduled	Participating patients who had their appointments reorganized during the pandemic had a 7.84-fold higher probability of missing an appointment (95% CI: 2.66, 23.08; p < 0.001)
Getting an appointment	Participating patients who had struggled to get an appointment during the pandemic were 31.50 times more prone to miss an appointment (95% CI: 4.32, 229.70; p = 0.001)

However, these findings should be deciphered with care in view of the small number of patients who missed their appointments as well as uncertainty due to the wide confidence intervals.

Availability of medicines before and after the pandemic from the patients’ perspective

Most participating patients before the pandemic stated that medications were freely accessible, with only one (0.4%) reporting poor availability of necessary medicines. A comparable situation was witnessed throughout the COVID-19 period, with only eight (3.3%) participating patients reporting they were hardly available, which was statistically significant (p < 0.001) (Table 7, building on Table 3). Of the participant patients, 53.1% reported no change in the prices of oncology medicines during the pandemic.

**Table 7:** Medication availability before and during the COVID-19 pandemic Note: All quoted parts denote reference [61].

“Medication availability”	“Before COVID-19, (n, %)”	“During COVID-19, (n, %)”	“p-value”
On every occasion	“40 (16.6%)”	“25 (10.4%)”	“<0.001”
Nearly all occasions	“87 (36.1%)”	“61 (25.3%)”	
Occasionally	“113 (46.9%)”	“147 (61.0%)”	
Very seldom	“1 (0.4%)”	“8 (3.3%)”	

Time series analysis of clinic attendance before and after the COVID-19 pandemic

**Figure 2:**
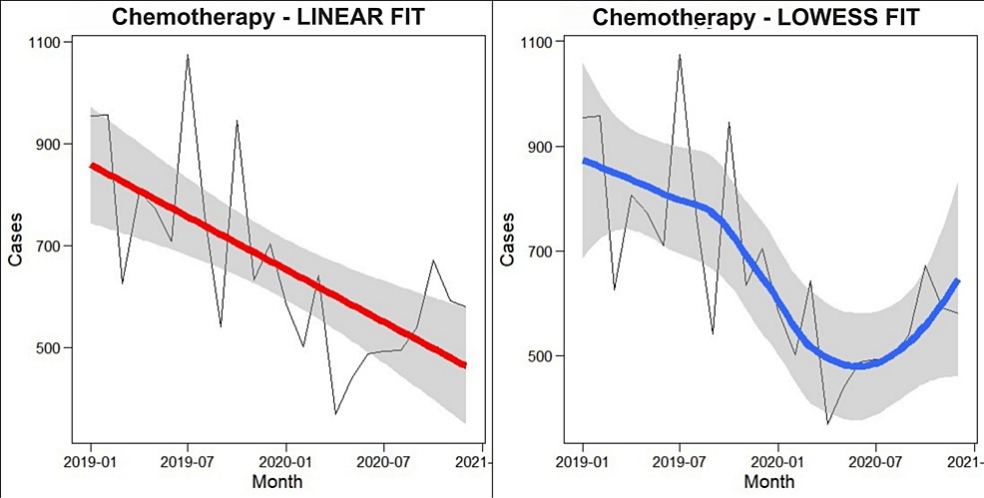
Clinic attendance before and after the COVID-19 pandemic

A decrease in attendance appears to have begun in the pre-COVID-19 era (January to December 2019) and sustained into the COVID-19 era (January to December 2020). The Lowess plot (Figure 2) showed that the rate of decline increased substantially afterward the COVID-19 lockdown restriction measures were put into place.

Chemotherapy administration attendance subsequently gradually increased following the strident deterioration during the COVID-19 lockdown period. This started rising again after July 2020, when the controlling measures were raised; however, the turnout endured poorer associated with analogous months in the previous year (2019).

**Figure 3:**
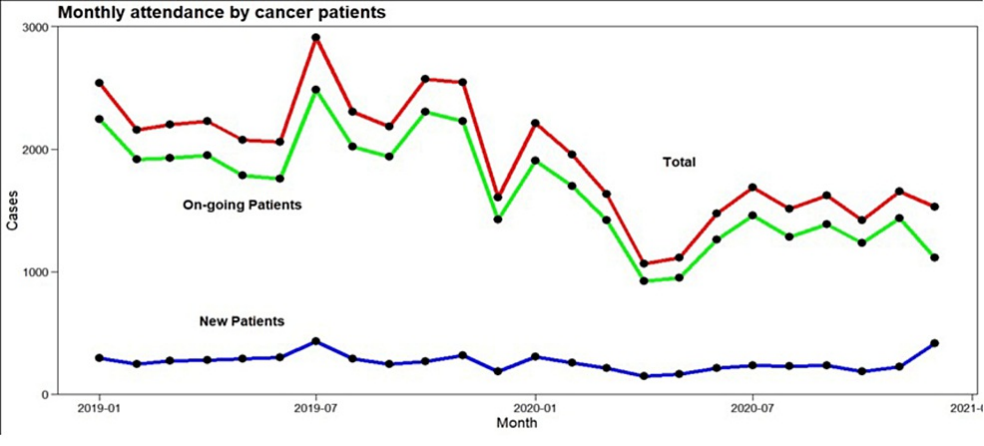
Monthly clinic attendance before and during the COVID-19 pandemic

Figure 3 shows the monthly attendance of existing and new patients at the oncology clinic and the total attendance before and during the pandemic. Peak attendance generally occurred in July; the lowest was observed in December 2019.

In January and February 2020, i.e., before the restrictions for COVID-19 were introduced, attendance was notably lower than in the previous year. In addition, from July 2019, there was a sturdy waning in repeat outpatient attendance even before COVID-19 restrictions were introduced. This decline endured and touched its nethermost point in April 2020, which applied to new cases and repeat visits from existing patients.

After April 2020, attendance enhanced, peaked in July, and remained constant until the end of 2020. Conversely, it was vibrant that after April 2020, presence was extremely worse paralleled to comparable months in the preceding year. There was petite seasonal changeability regarding freshly reported cases, with virtually incessant monthly attendance all around the year. Attendance of new cases of cancer patients reached its highest point in December 2020.

**Figure 4:**
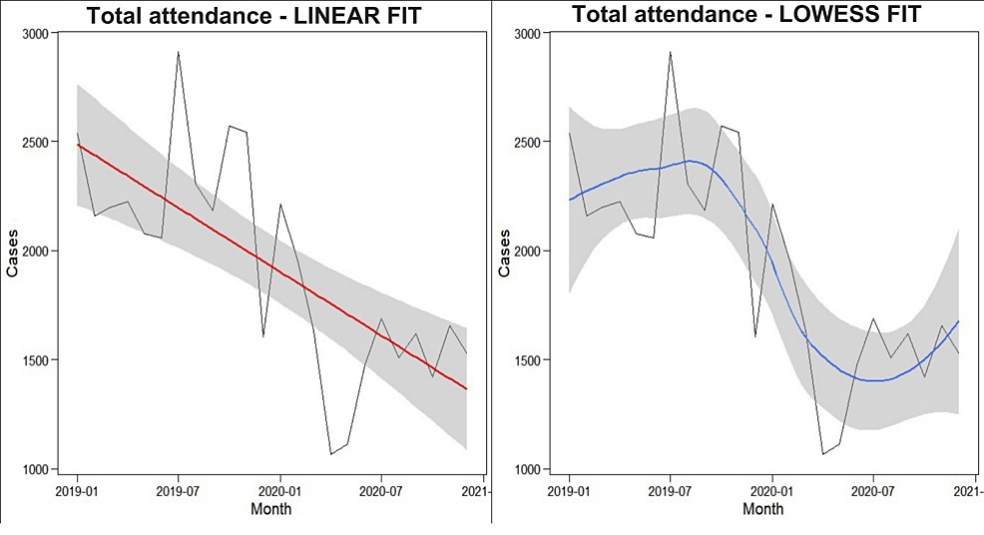
Trend in the outpatient attendance

Since total attendance and the attendance by existing patients were very similar, a trend line was only fitted for total attendance (Figure 4).

**Figure 5:**
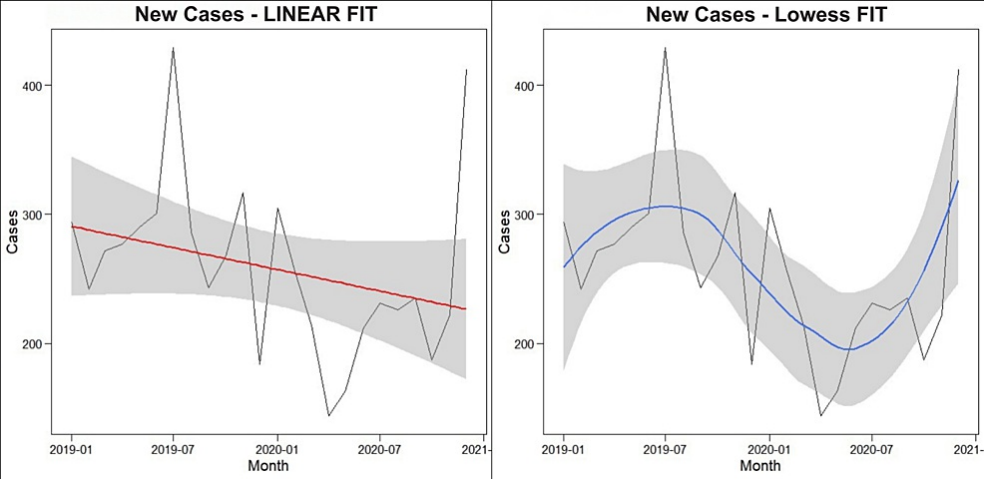
Trend in attendance of new cancer patients

The collaboration term of the out-patient attendance (p = 0.041) was statistically significant. This exhibited that mean attendance declined over time, with a distinctive change point in June 2020. Regarding existing patients, the interaction term was statistically insignificant (p = 0.121). According to the interaction term for the linear equation of new patients, it was statistically significant (p = 0.032), and the change point occurred in June 2020. Whilst mean attendances were decreasing, there was an overall increase in the number of new patients, with Figure 5 showing little seasonal variability [61].

## Discussion

This appears to be the first published study in Kenya to determine the impact of restrictions introduced at the onset of the COVID-19 pandemic to restrict its spread on out-patient attendance among cancer patients in Kenya and the subsequent utilization of chemotherapy services. These findings build on the dissertation of Njung’e IM [61], as well as the concerns of Kassaman et al. (2020) [45] and Umar et al. (2022) [58], which showed that issues of travel times and costs, alongside the restriction measures, had a profound impact on patients accessing cancer care during the pandemic [45,58].

Our findings showed that the utilization of scheduled chemotherapy services among participating cancer patients abridged noticeably in 2020, paralleled with the analogous period in 2019, with the lower off more noticeable from April to June 2020. This no doubt reflects the impact of the lockdown actions initiated by the Government in Kenya to control the spread of the virus, with no effective treatments or vaccines being available at the start of the pandemic [45,58,68]. Similar findings were seen in Morocco, where there was a 24% reduction in cancer case admissions from March to April 2020 when lockdown and other measures were implemented compared to 2019 [30]. Alongside this, the introduction of lock-down measures similarly affected the administration of chemotherapy across various countries [39,46,69-71]. The panic of contact with COVID-19, the restructuring of healthcare facilities, and economic barriers, including the increase in travel costs exacerbated by the loss of incomes, further affected patients’ attendance of chemotherapy appointments across countries, especially among LMICs [30,39,46,72,73].

**Figure 6:**
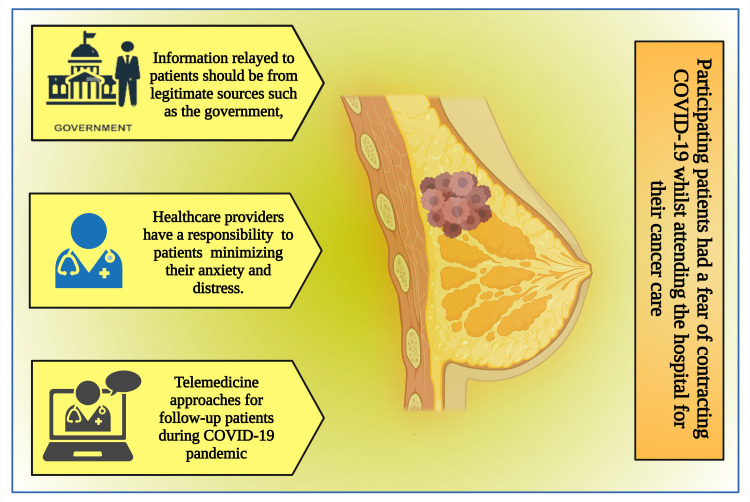
Showing schema of multiple approaches that were done to provide treatment to cancer patients during the COVID-19 pandemic Note: This figure has been drawn with the premium version of BioRender (https://biorender.com/ accessed on 8 November 2023) with license number CF262IWEB8. Image credit: Susmita Sinha.

A significant proportion of participating patients in our study also had a fear of contracting COVID-19 whilst attending hospital clinics for their cancer care. However, encouragingly, this fear did not appear to deter patients in our study from seeking chemotherapy services, which seems to be different from the findings in other studies [45,46,74]. Generally, healthcare providers should take responsibility for providing clear and essential information to cancer patients to minimize their anxiety and distress (Figure 6). Information provided to patients should be from authentic resources such as the national public agencies. This is particularly important during pandemics as there have been appreciable distorted reports and news regarding COVID-19 and its management, negatively impacting care delivery [64,75-77].

There has also been a rise in telemedicine approaches for follow-up across countries because of the COVID-19 pandemic and associated containment procedures and methods, in addition to reducing the financial burden on patients [39,54,55,72,73]. However, telemedicine was not widely adopted in our study, although a limited number of cases could consume telemedicine by making a phone call to their serving doctor. Having said this, this hybrid approach is likely to grow across countries, especially if it reduces the financial burden on patients as well as reducing the risk of immunocompromised patients acquiring infections from clinic attendance during successive waves of future pandemics.

Of interest was that clinic attendance declined in our study before the COVID-19 pandemic. Possible reasons for this include the death of patients whilst on therapeutic intervention, their remission on completion of therapy, actual delays in treatment, and/or issues of affordability among patients with transport costs [45,78,79]. Treatment delays can also be due to laboratory testing processes, uncommunicated changes in day-of-treatment orders, discrepancies in care plans, delays of doctor instructions, and privation of covering to furnish for treatment financial outlay [80]. Overall, “14.9% of patients missed at least one scheduled appointment between March 2020 and December 2020 when the COVID-19” [60] constraints were in place, with similar outcomes regarding the pervasiveness of missed chemotherapy appointments. Similar findings were seen by Jazieh et al. (2020) among 365 cancer centers across 54 countries [42]. This is a concern as missed appointments have the potential for greater morbidity and mortality if not adequately addressed [81,82].

Given that a significant proportion of patients missed chemotherapy sessions in our study, it is critical to examine participating patients' measures to mitigate against the effects of missed appointments and the rationale for escaping treatment. The principal reasons for missed visits in our study included an appreciable increase in travel fares, living on the outskirts of Nairobi, rearranged appointments, and difficulty getting an appointment. The introduction of curfews as well as social distancing regulations and travel bans from rural areas and other counties in Kenya to the principal county, Nairobi, resulted in an appreciable increase in fares [61]. The impact of this increase was confounded by the rise in unemployment during the pandemic; with this increase, it was detected to be a sovereign forecaster of missed chemotherapy appointments with an odds ratio of 4.56, like other studies in Kenya [45].

The locality of a participating patient’s physical residence was also important when lockdown restrictions were implemented in our study, with Nairobi, the capital city, in total lockdown. A significant proportion of patients in our study (74.6%) lived outside of Nairobi, which has implications for accessing cancer care alongside the impact of an appreciable increase in fares, like the findings from other studies [44,46,72]. Rescheduling appointments and difficulty getting to appointments were appreciably associated with missed chemotherapy sessions in our study, delaying timely access to cancer care, like other studies in Kenya and beyond [30,34,45,58,73]. The introduction of cancer care facilities closer to patients’ homes, especially for patients living in rural areas, along with new approaches to the care of patients, including telemedicine facilities for follow-up, should now be considered in countries, including Kenya, in preparedness for the next pandemic. We will continue to monitor the situation.

In our study, comorbidity or multi-comorbidity was also an independent predictor of missed chemotherapy appointments. Increased toxicity with chemotherapy treatments, frailty, and treatment delays do impact negatively on the use of chemotherapy, which may be a factor for missed appointments in our study [83-86]. Consequently, patients with cancer could benefit from a one-stop healthcare delivery point when being treated that takes into consideration their other health needs. In addition, introducing a prompt feedback mechanism helps address any concerns raised regarding the potential side effects of any prescribed medications, the diagnoses made, and the potential implications for them and their family.

Multivariate analysis in our study also suggested that the marital status of participating patients was an independent predictor for missed chemotherapy sessions, with those who were married more likely to miss chemotherapy appointments than unmarried patients. This appears to be counter-intuitive since married patients are presumed to lead more organized and planned lives, contrary to the findings of other studies [87]. However, a possible cause could be a lack of moral support from their partner, particularly if they do not have any illness themselves, versus family members. This will be explored further in future studies.

Limitations of this study

There are several limitations to our study. Firstly, the study was conducted in a single institution for the stated reasons, limiting the generalizability of the results. However, we believe this is less of an issue in Kenya given the current organization of public cancer services in the country similar to other African countries. Secondly, the study only focused on one form of cancer treatment and did not include all the other modalities used to manage cancer patients. However, chemotherapy is recognized as a cornerstone of managing patients with cancer. Thirdly, we used self-reported questionnaires. This though reflects concerns with record keeping in paper-based records, which is similar to other African countries. However, this is less of an issue here assisted by the comprehensive health records from the oncology department at KNH. There are always concerns with the accuracy of recall by patients and any associated bias. Fourthly, there are always concerns with time series analysis in view of the chosen time intervals. Fifthly, we are also aware that we did not explore further key advances such as telemedicine. However, despite these limitations, we believe the current findings are robust providing guidance for the future not only to key stakeholders in Kenya but also beyond to other African countries.

## Conclusions

In conclusion, our study found that the measures to address the COVID-19 pandemic and fears among patients negatively impacted the utilization of chemotherapy services among cancer patients at KNH. As a result, policymakers need to develop interventions that help mitigate the effects of the pandemic on the delivery of cancer care nationally and regionally in Kenya and other LMICs, with consideration of the long-term insinuations of the pandemic on the clinical outcomes of patients with cancer. This is important given the increasing priority for treating cancer patients across countries, given the increase in recent years with subsequent implications on the morbidity and mortality of patients. Based on our study findings and the implications, cancer treatment facilities should be increased in rural areas in Kenya and across Africa and other similar LMICs. Decentralizing current cancer facilities should positively impact the approachability or user-friendliness of cancer care among all patients and reduce the monetary encumbrance accompanying delivering cancer care in Kenya, especially with increased travel costs, and across LMICs.

Telemedicine is helping to revolutionize cancer care delivery by providing prompt feedback and follow-up support. Consequently, telemedicine is here to stay and should be widely adopted in Kenya. However, there must be safeguards to maintain the quality of care as well as provide safe and confidential interaction between healthcare professionals and patients. Furthermore, patients booked for telemedicine consultations should be appraised on a case-to-case basis. Telemedicine also requires the necessary facilities in patients’ homes, which was not always the case at the start of the pandemic. Other suggested measures in preparedness for future pandemics include the need to adjust the dosing schedules for chemotherapy as well as replace IV with oral alternatives where possible. Such actions should diminish hospital visits while safeguarding cancer patients from travel and clinic attendance vulnerabilities during future pandemics. In addition, it enhances the possibility of telemedicine consultations. Nonetheless, these adjustments should have pertained on a case-by-case basis, and any changes in medicines prescribed or administration routes altered should be comprehensively assessed so as not to make concessions to the beneficial therapeutic effect for patients. Reorganizing healthcare possessions and supplies, including the potential delivery of other health services together with cancer care in patients with comorbidities, should help improve their subsequent care. The well-being of patients with cancer also needs to be prioritized going forward, given increasing rates, especially if patients have fears about clinic attendance and/or concerns that their diagnosis and treatment are being upset by movement control orders and other procedures. Public health messaging regarding the prevention and management of patients with cancer during the pandemic is also essential, given the extent of misinformation regarding COVID-19 that was seen in practice. Pertinent use of social media could also help here.
